# Seroprevalence and Associated Outcomes of Parvovirus B19 Infection in Human Immunodeficiency Virus Patients: A Systematic Review

**DOI:** 10.1055/s-0045-1801865

**Published:** 2025-01-27

**Authors:** Sagad O. O. Mohamed, Reem A. A. Mohamedelmugadam, Safa A. M. Almardi, Tassnem H. M. Ahmed, Malaz E. H. Ibrahim, Abdalla O. O. Mohamedali

**Affiliations:** 1 Department of Pediatrics and Child Health, Faculty of Medicine, University of Khartoum, Khartoum, Sudan; 2 Institute of Endemic Diseases, Faculty of Medicine, University of Khartoum, Khartoum, Sudan; 3 Department of Internal Medicine, Faculty of Medicine, University of Khartoum, Khartoum, Sudan; 4 Department of Internal Medicine, Faculty of Medicine, Shendi University, Shendi, Sudan

**Keywords:** human immunodeficiency virus, HIV, anemia, parvovirus B19, PVB19

## Abstract

Several case reports have highlighted the presence of serious clinical outcomes in patients with human immunodeficiency virus (HIV) related to parvovirus B19 (PVB19). However, epidemiological studies have produced inconsistent and varying results regarding the prevalence of PVB19 and its associated clinical outcomes in this population. These inconsistencies highlight the need for a thorough summary and analysis of present data to better understand burden and impact of PVB19 on HIV patients. This review aims to provide an overview of current evidence and identify areas for further research.

Following the Meta-analyses of Observational Studies in Epidemiology (MOOSE) guidelines, a comprehensive search was conducted across Medline/PubMed, Google Scholar, and World Health Organization Virtual Health Library Regional Portal. The pooled prevalence with the corresponding 95% confidence interval (CI) was measured using Comprehensive Meta-Analysis Software version 3.3. Publication bias was estimated based on Begg's test, Egger's test, and examination of the funnel plots.

A total of 16 studies, with 2,122 HIV patients, were included in the meta-analysis. The pooled prevalence of detecting anti-PVB19 immunoglobulin G, anti-PVB19 immunoglobulin M, and PVB19 DNA particles among HIV patients was 43.6% (95% CI: 23.5–66.1%), 5.10% (95% CI: 2.10–12.10%), and 6.40% (95% CI: 4.10–9.90%), respectively. In the overall population of HIV patients, most of the included studies did not establish a statistically significant association between PVB19 infection and the occurrence of anemia.

PVB19 infection is commonly detected in individuals with HIV. However, anemia due to PVB19 is not common in this population. Findings from a few studies suggest that PVB19 infection may contribute to anemia in individuals with advanced HIV disease or significant immunosuppression. Additional research is needed to confirm and clarify these relationships in individuals with HIV, particularly those with compromised immune systems.

## Introduction

Human immunodeficiency virus (HIV) is one of the most important retroviruses worldwide, especially HIV-1, as it causes acquired immunodeficiency syndrome (AIDS). The virus infects and destroys CD4 T-cells, attenuating cell-mediated immunity and leaving the host vulnerable to clinical manifestations, including a variety of opportunistic infections and cancers. 1 2

Anemia is the most common hematologic abnormality associated with HIV infection and prevalence and severity of anemia increase as the HIV infection advances to AIDS. Varying causes of anemia have been described in HIV patients. 3 4 5 These include direct hematological effects of HIV infection itself within the marrow microenvironment, malnutrition especially vitamin B12 and iron deficiency, opportunistic infections by *Mycobacterium tuberculosis, Pneumocystis jiroveci* , and the use of zidovudine, a commonly used Highly Active Antiretroviral Therapy (HAART) for management of HIV infection. 3 4 5 One of the recognized causes of anemia is parvovirus B19 (PVB19), a small nonenveloped virus of a single-stranded DNA, due to its ability to affect primary erythroid progenitors, resulting in persistent infection and chronic anemia. 6 7 8

Infection with PVB19 had been reported globally, and it is most commonly transmitted as droplet infections through the respiratory secretion, or vertically through the placenta to the fetus, and through bone marrow and organ transplantations. 7 8 This virus can lead to self-limited symptoms in healthy individuals. However, PVB19 infection can be involved in several clinical manifestations, such as arthropathy, erythema infectiosum, chronic arthritis, hydrops fetalis, and transient aplastic crisis in patients with sickle cell anemia and pure red cell aplasia in immune compromised hosts. 7 8 9

Common methods for diagnosing PVB19 infection include serological tests that detect antibodies to the virus's capsid proteins (VP1 and VP2) and other tests that detect viral DNA particles. Antibody detection methods include serum-specific immunoglobulin G (IgG) antibody testing, used to confirm past exposure to PVB19, and serum immunoglobulin M (IgM) antibody testing, used to confirm acute PVB19 infection. Additional diagnostic tools include viral DNA detection using the polymerase chain reaction technique. 7 8 10

While several case reports highlighted the presence of serious clinical outcomes in HIV patients related to PVB19 infection, epidemiological studies have produced inconsistent and varying results regarding the prevalence of PVB19 and its associated clinical outcomes in this population. These inconsistencies highlight the need for a thorough summary and analysis of present data from these studies to achieve a more accurate understanding of PVB19 burden in HIV patients. This review aims to provide a comprehensive overview of the current evidence and identify areas requiring further investigation.

## Methods

### Search Approach and Study Inclusion Criteria

The methodology for this review adhered to Meta-analyses of Observational Studies in Epidemiology (MOOSE) guidelines. To gather relevant literature, we conducted a systematic literature search using the electronic databases of MEDLINE (via PubMed), World Health Organization Virtual Health Library Regional Portal, and Google Scholar. The search covered all available literature from its inception up to June 2024. There were no restrictions applied to the search in terms of geographical area or publication date.

The search terms used were based on combining Mesh terms and keywords to insure no possible relevant articles were missed. Furthermore, to identify additional studies for inclusion in this review, we conducted a manual search by carefully screening the reference lists of the studies already included. The publications that were found were uploaded to Rayyan software (Qatar Computing Research Institute, Doha, Qatar; http://rayyan.qcri.org ) to expedite initial screening of titles and abstracts and remove duplicate entries. 11

The search terms used for B19V were (((Parvoviridae[Mesh] OR Parvovirus[Mesh] OR Parvovirus B19[Mesh] OR parvovirus b19, human[MeSH Terms] OR Parvovirus[tw] b19[tw] OR Parvoviridae[tw] OR B19V[tw] OR PVB19[tw] OR “B19 virus”[tw] OR Erythrovirus[tw] OR “Erythema infectiosum”[tw] OR “Fifth disease”[tw] OR “slapped cheek disease”[tw] OR “slapped cheek syndrome”[tw]).

Regarding HIV, the search terms used were (((HIV Infections[MeSH] OR HIV[MeSH] OR Acquired Immunodeficiency Syndrome[Mesh] OR hiv[tw] OR hiv-1*[tw] OR hiv-2*[tw] OR hiv1[tw] OR hiv2[tw] OR hiv infect*[tw] OR “human immunodeficiency virus”[tw] OR “human immunedeficiency virus”[tw] OR “human immuno-deficiency virus”[tw] OR “human immune-deficiency virus”[tw] OR ((“human immun*”)[tw] AND (“deficiency virus”) [tw]) OR “acquired immunodeficiency syndrome”[tw] OR “acquired immunedeficiency syndrome”[tw] OR “acquired immuno-deficiency syndrome”[tw] OR “acquired immune-deficiency syndrome”[tw] OR ((“acquired immun*”[tw]) OR (“deficiency syndrome”[tw]))))).

### Inclusion and Exclusion Criteria

The inclusion criteria for the articles in this review included cross-sectional, case-control, or cohort studies that provided explicit data on the number of HIV patients with PVB19 infection. Infection was determined by the presence of positive anti-PVB19 IgG, anti-PVB19 IgM, or PVB19 DNA particles.

We excluded case reports, editorials, reviews, abstracts, and studies that did not provide sufficient data on the variables of interest. To ensure a better representative sample, we excluded studies that focused solely on specific subsets of the HIV population. Therefore, we excluded studies that only included individuals with either chronic or severe anemia, as well as studies that only included healthy individuals without any symptoms.

First, the initial screening process involved reviewing titles and abstracts to identify potential studies for inclusion. Subsequently, the full-text articles were then thoroughly reviewed to confirm their eligibility based on the predefined criteria.

### Quality Assessment and Data Extraction

Quality evaluation of the included studies was done using the Joanna Briggs Institute critical appraisal checklists ( https://jbi.global/critical-appraisal-tools ). These tools were set to assess the methodological quality of studies and to determine to what extent a study addressed the possibility of bias in study design, conduct, and data analysis. Data were extracted from each study by three independent reviewers, and any discrepancies among them was resolved by discussion and consensus.

### Statistical Analysis

The statistical analyses were performed by using Comprehensive Meta-Analysis Software version 3.3 (Biostat, Engle-wood, New Jersey, United States; http://www.Meta-Analysis.com ). The heterogeneity was assessed through I ^2^ test, which describes the percentage of variability in the effect estimates. In instance of high heterogeneity, the pooled summary prevalence was calculated using random-effects models. 12 Publication bias was assessed using Begg's test, Egger's test, and visual inspection of the funnel plot. 13 If publication bias was detected, the Duval and Tweedie trim and fill method was employed to account for potential missing studies and calculate the adjusted pooled estimate. 14

## Results

### Study Characteristics

**Fig. 1:**
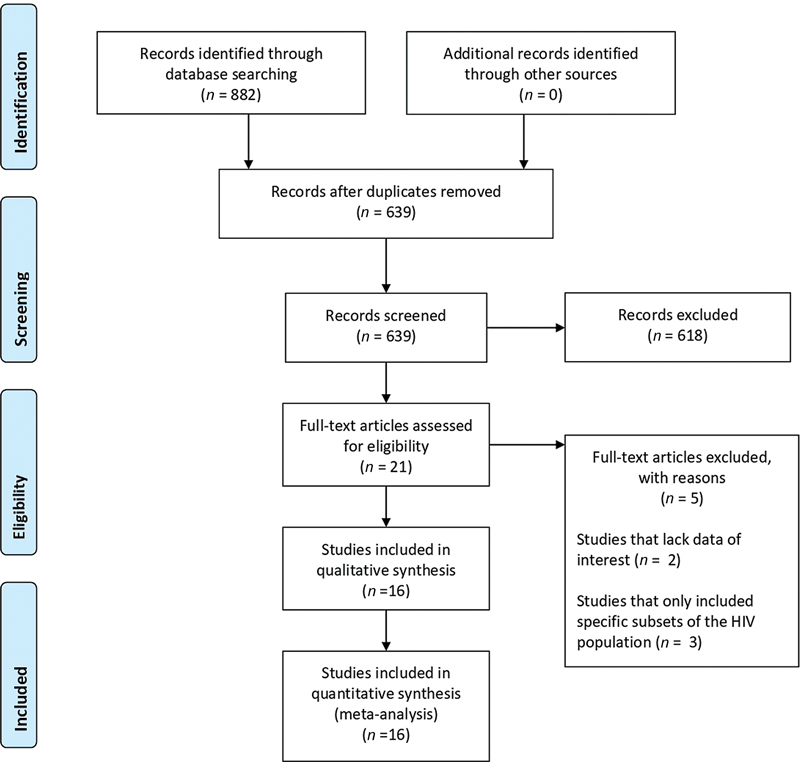
Flow chart for studies' selection process.

The schematic flow of the study identification and selection process is presented in Fig. 1 . Initially, the search yielded a total of 882 records. After removing duplicate data (243 articles), 639 studies were included for the title and abstract screening. Of which, 618 were excluded due to irrelevance or based on the exclusion criteria. Full texts of the remaining 21 records were screened with a subsequent exclusion of 5 records as shown in Fig. 1 . Lastly, a total of 16 studies published from 1992 to 2020 met the eligibility criteria and were further included for the analyses. 5 9 15 16 17 18 19 20 21 22 23 24 25 26 27

The majority of the studies primarily focused on adult populations, with only three studies including children with HIV. Anemia was the most common clinical abnormality assessed by the included studies. Additionally, only four studies examined the genotypes of PVB19 isolates, with genotype 1 being the only identified across all except one study, which reported the presence of genotypes 2 and 3 as well. The main features and summary of the included studies are presented in Tables 1 and 2 .

**Table 1:** Baseline characteristics of the studies included in the review

Study	Publication year	Country	Study type	Age group	Quality assessment (risk of bias)
Nigro et al 35	1992	Italy and Romania	Cross-sectional	Children	6
Chernak et al 22	1995	United States	Cross-sectional	Adults	8
Kerr et al 19	1997	Ireland	Case-control	Adults	5
Aguiar et al 26	2001	Brazil	Cross-sectional	Adults	5
LaMonte et al 17	2004	United States	Cohort	Children	7
Compston et al 21	2009	Ghana	Cross-sectional	Adults	8
He et al 20	2012	China	Cross-sectional	Adults	5
Avezedo et al 23	2012	Brazil	Cross-sectional	Adults	8
Abdollahi et al 27	2014	Iran	Case-control	Adults	7
Azadmanesh et al 24	2015	Iran	Cross-sectional	Adults	6
Shrikhande et al 15	2017	India	Cross-sectional	Adults	5
Pedranti et al 16	2017	Argentina	Case-control	Adults	5
Kiani et al 18	2018	Iran	Cross-sectional	Adults	5
Aleru et al 25	2018	Nigeria	Cross-sectional	Children	5
Nouri et al 9	2019	Iran	Case-control	Adults	6
Ashaka et al 5	2020	Nigeria	Case-control	All	6

**Table 2:** Summary of the studies included in the review

Study	No. of HIV patients	Anti-PVB19 IgG	Anti-PVB19 IgM	PVB19 DNA particles	Other findings
Nigro et al 35	71	48	18	–	PVB19 infection was associated with anemia in the Italian participants.
Chernak et al 22	127	12	0	2	Patients with high viral load of PVB19 had lower CD4 count and high rate of anemia. No statistical test of significance was done.
Kerr et al 19	61	–	–	13	Patients with PVB19 infection had lower CD4 count and hemoglobin levels. No statistical test of significance was done.
LaMonte et al 17	91	Not specified a	Not specified a	3	No statistically significant association with anemia.
Compston et al 21	296	212/286	–	0/296	No significant relation with symptomatic HIV condition.
He et al 20	573	–	–	26	The three genotypes of the virus were detected. No clinical associations were investigated.
De Avezedo et al	88	28	3	4	Higher odds of anemia among those with positive PVB19 IgG.
Abdollahi et al 27	90	73	26	–	No statistically significant association with anemia.
Azadmanesh et al 24	99	11	1	13	All sequenced B19 isolates belonged to genotype 1. No statistically significant association with anemia.
Shrikhande et al 15	100	11	6	–	No statistically significant association with CD4.
Pedranti et al 16	98	66	5	15	Patients with PVB19 infection had lower CD4 count. No statistically significant association with anemia.
Kiani et al 18	100	–	–	10	No clinical associations were investigated.
Aleru et al 25	158	–	–	2	All sequenced PVB19 isolates belonged to genotype 1. The two PVB19-positive children had moderate to severe anemia.
Nouri et al 9	113	19	3	7	All sequenced PVB19 isolates belonged to genotype 1. Higher PVB19 viral load was associated with lower CD4 count and anemia.
Aguiar et al 26	55	50	0	6	No statistically significant association with anemia.
Ashaka et al 5	200	–	–	13	No statistically significant association with anemia.

Abbreviations: HIV, human immunodeficiency virus; IgG, immunoglobulin G; IgM, immunoglobulin M; PVB19, parvovirus B19.

a The study reported that 12 individuals tested positive for either recent or previous infection (IgG or IgM antibodies), but did not specify the exact number of individuals positive for each type of antibodies.

### Prevalence of PVB19 Infection among HIV Patients

**Fig. 2:**
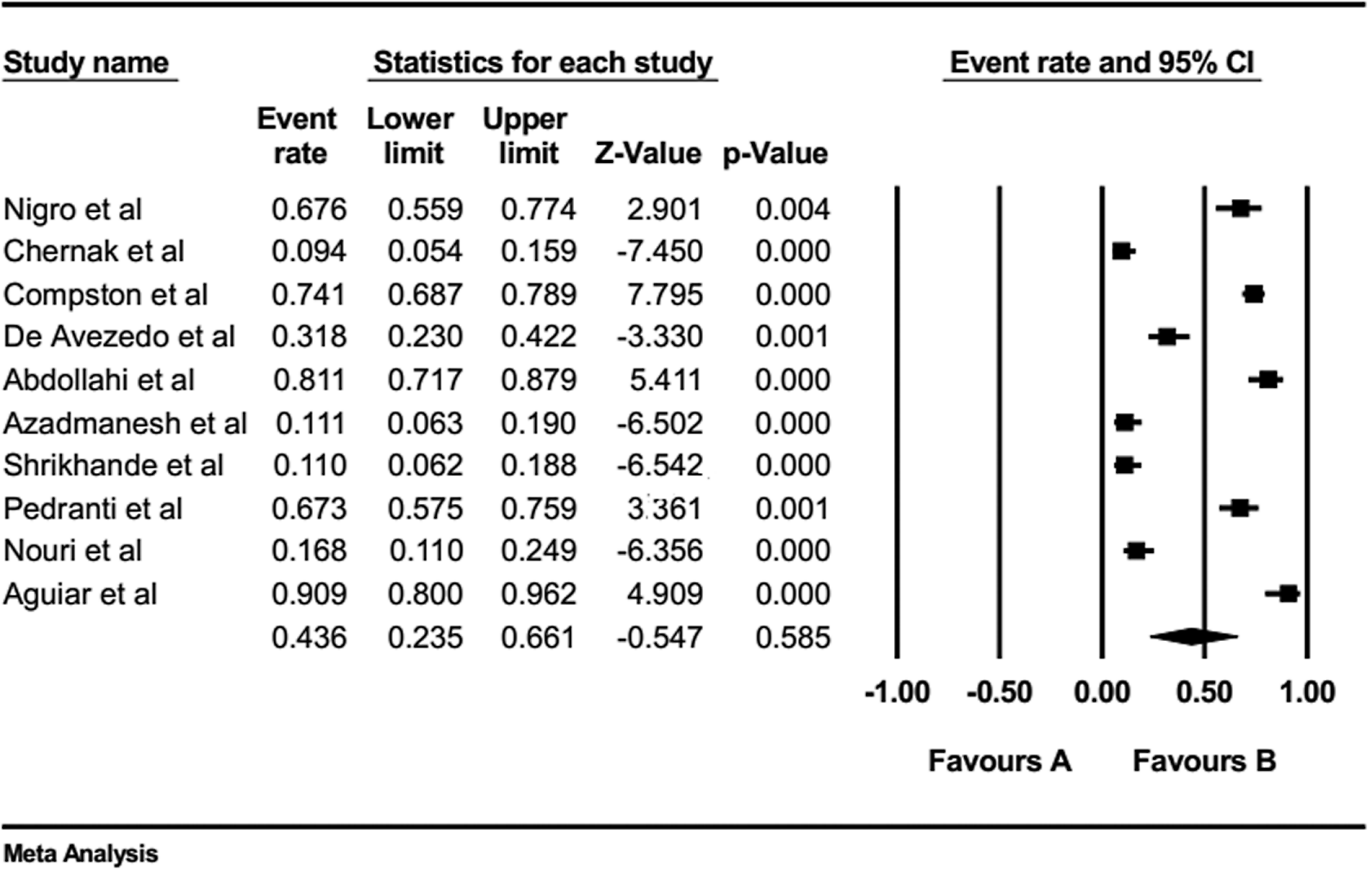
Seroprevalence of PVB19 IgG antibodies in patients with HIV. HIV, human immunodeficiency virus; IgG, immunoglobulin G; PVB19, parvovirus B19.

The meta-analysis for the included studies showed that the overall seroprevalence of anti-PVB19 IgG among HIV patients was 43.6% (95% confidence interval [CI]: 23.5–66.1%; Fig. 2 ). No evidence of publication bias was detected based on visual examination of the funnel plot and from the results of Begg's test ( *p* = 0.18) and Egger's test ( *p* = 0.09).

**Fig. 3:**
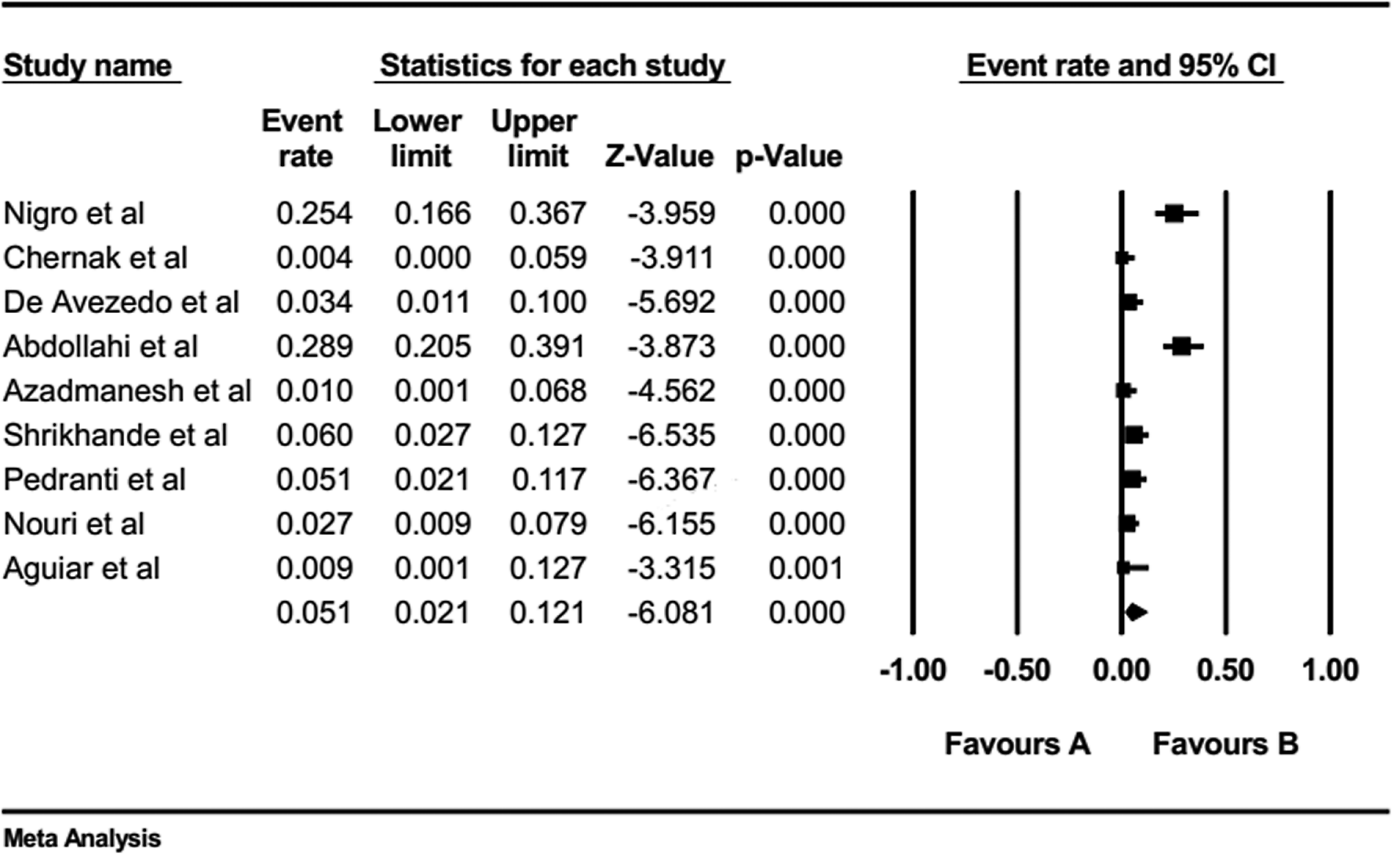
Seroprevalence of PVB19 IgM antibodies in patients with HIV. HIV, human immunodeficiency virus; IgM, immunoglobulin M; PVB19, parvovirus B19.

The seroprevalence of anti-PVB19 IgM among HIV patients was 5.10% (95% CI: 2.10–12.10%; Fig. 3 ). The publication bias was significant for the Egger's test ( *p* < 0.01) but not for the Begg's test ( *p* = 0.08). However, upon applying the Duval and Tweedie trim and fill method to account for potential missing studies, no missing studies were identified, and the adjusted prevalence estimate remained similar to the initial finding.

**Fig. 4:**
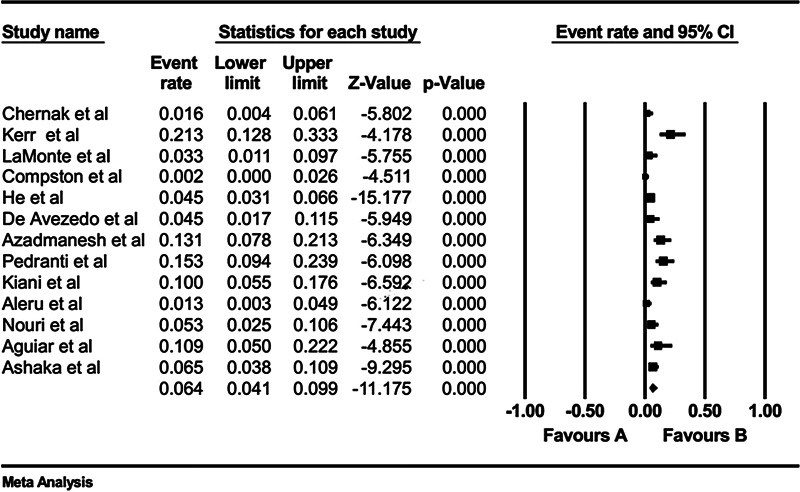
Prevalence of PVB19 DNA particle detection in patients with HIV. HIV, human immunodeficiency virus; PVB19, parvovirus B19.

**Fig. 5:**
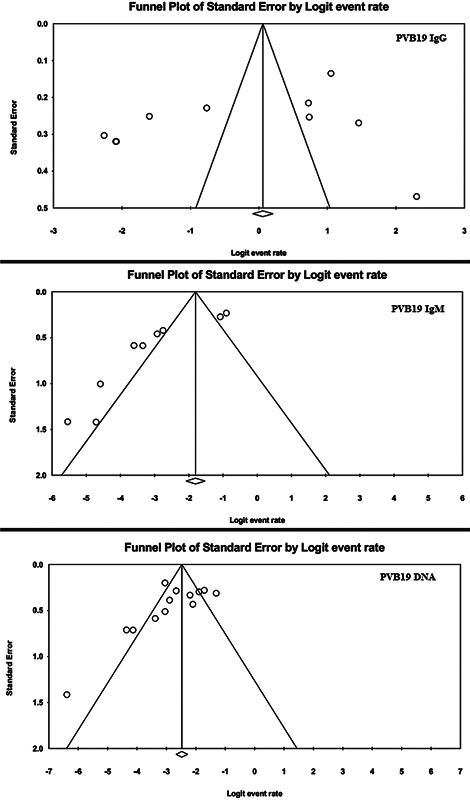
Funnel plot showing evidence of publication bias among studies in the meta-analysis of seroprevalence of PVB19 DNA particles, IgM antibodies, and IgG antibodies in patients with HIV. HIV, human immunodeficiency virus; IgG, immunoglobulin G; IgM, immunoglobulin M; PVB19, parvovirus B19.

Regarding results of PVB19 DNA detection among HIV patients, the pooled prevalence was 6.40% (95% CI: 4.10–9.90%; Fig. 4 ). The publication bias test was significant for the Begg's test ( *p* = 0.01) but not for the Egger's test ( *p* = 0.07). However, the Duval and Tweedie trim and fill method showed that no potential studies are missing, and the adjusted estimate was still significant and similar to the original findings ( Fig. 5 ).

### Correlation between PVB19 Infection and Anemia among HIV Patients

Most of the included studies assessed the association between PVB19 infection and anemia ( Table 2 ). Significant heterogeneity was observed in how these studies evaluated the correlation between HIV and anemia. This variability arose from differences in definitions and measures of anemia and HIV disease severity, such as CD4 count cut-points, viral load, and clinical staging. Due to this heterogeneity, a meta-analysis on the correlation between PVB19 infection and anemia was not feasible.

The review revealed that most studies did not report a statistically significant association between PVB19 infection and anemia in HIV-positive individuals ( Table 2 ). However, a few studies showed that PVB19 infection might contribute to anemia in specific subgroups, particularly those with advanced HIV disease or significant immunosuppression. Pedranti et al and Ashaka et al reported that anemia was predominantly observed in individuals with a CD4 count below 200 cells/μL. Similarly, Nouri et al found that patients with higher PVB19 viral loads tended to have lower CD4 counts and higher rates of anemia. 5 9 16

## Discussion

This meta-analysis found that 43.6, 5.10, and 6.40% of HIV patients had anti-PVB19 IgG, anti-PVB19 IgM, and PVB19 DNA, respectively. The relatively low prevalence of anti-PVB19 IgM found in the current meta-analysis is likely due to the fact that most included studies focused on adult populations, while primary or acute PVB19 infections are more common during childhood. 16 The pooled prevalence of PVB19 DNA, anti-PVB19 IgM, and anti-PVB19 IgG among HIV patients, as observed in this review, is slightly higher than, but generally comparable to, the findings of two recent meta-analyses that estimated the prevalence of PVB19 infection in the healthy population. 28 29

The variation in the reported prevalence of PVB19 among the included studies can be attributed to various factors that influence its presence in this population. These factors include the seasonal variation as well as specific characteristics of the study populations, such as their geographic location, age distribution, stage of HIV infection, and treatment status. 7

Regarding PVB19-related anemia, many of the included studies indicate a slightly higher proportion of PVB19 infection in anemic HIV patients compared with nonanemic patients. Nevertheless, this finding did not reach the statistical significance among most of these studies, which indicates that PVB19-related anemia among HIV patients is generally uncommon. However, a plausible explanation for this lack of significance is that these studies covered a broad range of HIV patients, including many who were asymptomatic or had mild immunosuppression, rather than specifically focusing on those with advanced HIV disease or profound immunosuppression.

The results of studies conducted by Pedranti et al and Ashaka et al indicate that there is evidence to suggest that parvovirus PVB19 may contribute to the development of anemia in HIV patients who have severe or profound immune suppression. 5 16 In addition, Nouri et al found that patients with higher PVB19 viral load had lower CD4 count and had higher rate of anemia, which suggest that PVB19 can replicate at a high level in those with severe immune suppression. 9 Likewise, it has been reported that PVB19-induced anemia is most commonly seen in immunosuppressed individuals who fail to produce sufficient antibody titers to effectively combat the PVB19 infection, such as recipients of solid organ transplants, individuals undergoing chemotherapy treatment, and those with congenital immunodeficiency disorders. 7 30

Some of the reviewed studies proposed that patients with advanced HIV who have severe immune suppression may have lacked the ability to produce detectable antibody to PVB19. 9 20 23 26 The advanced stages of HIV are characterized by diminished immune response with antibodies production less effective in controlling infections. 16 This impaired immunity status leads to persisting PVB19 infection, resulting in chronic anemia. 6

In addition to the effect of immune status, low burden of hematological disorders observed in this review among the general population of HIV patients can be explained by the widespread use of HAART. Use of HAART has been shown to stimulate reconstitution of humoral and cell-mediated immune competence to various opportunistic infections. 3 31 Therefore, with the advent of widespread effective HAART therapy, much of the burden of severe hematological changes caused by PVB19 in HIV patients has been reduced. 30 32

While some studies in the literature often highlight the presence of severe anemia caused by PVB19 infection among HIV patients, these studies are often limited to case reports or case series, making it difficult to generalize their findings to the broader HIV population. In addition, some of these reports involved patients who were not receiving HAART therapy or were done before widespread availability of HAART. 31 33 As a result, these publications may give the impression that PVB19 is a major risk factor for severe anemia in the entire HIV patients. 16

However, because persistent PVB19 infection is a treatable cause of anemia through the administration of intravenous immunoglobulin, as well as adherence to HAART, PVB19 infection should continue to be considered in the differential diagnosis of anemia in HIV infected patients, especially when the patients have chronic or severe anemia that cannot be explained otherwise. 4 17 20 31 Several cases of pure red cell aplasia secondary to PVB19 has been described and showed significant improvements after initiation of treatment. 4 30 33 34

Our review identified several research gaps and unanswered questions that need to be addressed. Data on the distribution of PVB19 infection on HIV patients are limited, especially in different geographical regions and populations. There is limited understanding of influence of different HIV stages and HAART regimens on impact of PVB19 infection in HIV patients. The spectrum of clinical manifestations associated with parvovirus infection in HIV patients was not fully studied in large-scale studies or longitudinal studies. In addition, research on PVB19 infection in HIV-infected pregnant women is limited, particularly regarding fetal development and maternal health. While some studies have investigated PVB19 genotypes, the role of host genetic factors in susceptibility to PVB19 infection and its complications remains largely unexplored. Addressing these research gaps is essential for improving the understanding, diagnosis, and management of PVB19 infection in HIV patients and ultimately improving their health outcomes.

The key limitations of this review are heterogeneity of the included studies and the limitations in the available data. These restrict our ability to conduct additional meta-analyses to explore other aspects of the PVB19 infection in HIV patients beyond estimating the overall prevalence. Additionally, the inclusion of only English-language publications may limit overall representativeness of the findings of this review.

## Conclusion

PVB19 infection is commonly detected in individuals with HIV. However, anemia due to PVB19 is not common in this population. However, findings from a few studies suggest that PVB19 infection may contribute to anemia in individuals with advanced HIV disease or significant immunosuppression. Additional research is needed to confirm and clarify these relationships in those with severe immune suppression.
